# Detection of Quadruplex DNA by Gold Nanoparticles

**DOI:** 10.1155/2012/327603

**Published:** 2012-03-27

**Authors:** Heather F. Crouse, Alex Doudt, Cassie Zerbe, Swarna Basu

**Affiliations:** Department of Chemistry, Susquehanna University, 514 University Avenue, Selinsgrove, PA 17870, USA

## Abstract

Gold nanoparticles have been used as a probe to detect low (<10 ppb) concentrations of quadruplex DNA. These nanoparticles display a tendency to form aggregates in the presence of certain quadruplex forms, as observed via enhanced plasmon resonance light scattering (PRLS) signals. These nanoparticles showed differing degrees of interactions with different types of quadruplex and mixed sequences but no interaction with duplex DNA. Enhancement of PRLS signals greater than 50% was observed at nanomolar DNA concentration, and a lower limit of detection of 2.1 nM was established for three different quadruplex DNA sequences, including the thrombin-inhibiting single-stranded 15 mer aptamer DNA, d(GGTTGGTGTGGTTGG), and the double-stranded 12 mer DNA, d(G4T4G4). Two different sample preparation protocols were used for the PRLS experiments, and they yielded similar results.

## 1. Introduction

Cost-effective and efficient methods for the selective detection of quadruplex structures present in many structural forms of DNA have been difficult to develop. A number of techniques have been used to monitor quadruplex formation. These techniques include, but are not limited to, nuclear magnetic resonance (NMR) spectroscopy, circular dichroism (CD), Raman spectroscopy, and absorption and fluorescence spectroscopy [1–6]. These methods consume large quantities of DNA, require expensive apparatus, or require elaborate sample preparation. Thus, there has been a lot of interest in the development of novel methodologies and probes for rapid and reliable detection of trace amounts of quadruplex DNA. Our research group has shown that a terbium chelate can detect small (20 ppb) amounts of both single-stranded and double-stranded quadruplex DNA and that the chelate might be binding to the DNA [7]. In this work, the ability of gold nanoparticles to detect low concentrations of DNA and distinguish between different quadruplex sequences is presented.

Nanoparticles are a suitable probe for DNA detection due to their small size, optical and magnetic properties. A variety of biological applications, including drug and gene delivery, biodetection of pathogens, tissue engineering, and tumor destruction via heating, have been developed [8–10]. One important optical property that nanoparticles display is the ability to efficiently scatter light. Colloidal gold nanoparticles are known to display strong plasmon absorption bands due to electron oscillations induced by the incident light [11–13]. These strong absorption properties result in gold colloidal suspensions displaying intense colors. In the presence of cations, aggregation of gold nanoparticles occurs, which causes a new red-shifted plasmon absorbance. Resonance light scattering occurs when the incident beam is at an energy similar to the absorption band produced by an oscillating dipole. The effect is amplified when two or more dipoles are strongly coupled [13]. This method holds the most promise as it provides a cost-effective and precise means of detecting quadruplex DNA under biologically important conditions and also provides insight into the nature of interactions between quadruplex DNA and nanoparticles. A recent study found that gold nanoparticle/quadruplex DNA suspensions display aggregation tendencies that give enhanced light scattering signals of the nanoparticles [11].

Quadruplex DNA is higher-order DNA structures that is formed from guanine-rich (G-rich) nucleotide sequences. These structures are comprised of stacked tetrads, each of which arises from the planar association of four guanines in a cyclic Hoogsteen hydrogen-bonding arrangement [14]. Quadruplex structures can be formed from one, two, or four separate strands of DNA that acquire a wide variety of different topological conformations [15]. A single G-rich repeat within a DNA sequence can form a tetramolecular parallel quadruplex. DNA sequences that contain two or more G-rich repeats have been shown to form G-G hairpins, which in turn dimerize to form multiple types of stable bimolecular quadruplexes. DNA sequences with four G-rich repeats can fold upon themselves to form an antiparallel intramolecular quadruplex [14].

Quadruplex structures have been widely studied due to their physiological importance. They have been identified in G-rich eukaryotic telomeres at the ends of eukaryotic chromosomes [15, 16]. Repetitive telomere sequences cap the eukaryotic chromosome, protecting the ends of the chromosome from damage and recombination. Telomerase is a ribonucleoprotein that elongates the G-rich strand of telomeric DNA and is reactivated in approximately 85% of tumors, contributing to their immortality [17]. The inhibition of Telomerase which is brought about by the formation of quadruplex structures has become an attractive and promising strategy for the development of an anticancer therapy. Small molecules that bind to and stabilize quadruplex DNA have also been shown to be effective Telomerase inhibitors [18]. Recently, quadruplex sequences have shown promise as novel antiretroviral gene therapies [19].

Monovalent cations, such as K^+^ and Na^+^, have been shown to stabilize these DNA quadruplex structures, presumably by coordinating with the eight carbonyl oxygen atoms present between stacked tetrads [20–24]. The presence of potassium also plays an important role in the formation of quadruplex DNA. The “chair” and “edge” type quadruplex structures require potassium to fully form and adopt the most stable structure [2, 25–27]. The “chair” type 15 mer quadruplex DNA is known to bind and inhibit Thrombin, an enzyme that facilitates blood clotting [2, 25, 28]. Quadruplex DNA may also play a role in chromosomal alignment and recombination, and the triplet repeat (GGC)_*n*_ sequence is associated with Fragile X syndrome, a DNA-replication-associated disease [29–31]. Quadruplex DNA can also form from two strands, and the 12 mer DNA forms a “basket” type quadruplex structure and does not require potassium to fully form [23].

In this study we have used gold nanoparticles as a low-concentration, biocompatible probe to detect small amounts (8 ppb) of quadruplex DNA, and we have tested different sample preparation protocols with the goal of demonstrating that nanoparticles can be used with a simplified sample preparation protocol making them a “ready to use” probe. This is important when developing protocols for *in vivo* applications. This procedure has been used to distinguish between various forms of quadruplex DNA and DNA that exist in “mixed” forms.

## 2. Experimental

### 2.1. Reagents and Chemicals

Duplex calf thymus DNA was obtained from Sigma-Aldrich. Quadruplex DNA sequences were custom-synthesized by Integrated DNA Technologies (Coralville, IA). All DNA sequences were used without further purification. DNA stock solutions were prepared in HEPES buffer (20 mM HEPES, 140 mM NaCl at pH 7.0). DNA concentrations were verified using the following extinction coefficients at 260 nm: 12 mer 115,200, d(GGC)_3_ 81,300, 15 mer 143,300, 17 mer 156,900. DNA purity was checked by measuring the ratio of absorbances (A_260_/A_280_). Potassium chloride was added as needed to ensure a 5 mM KCl concentration in solution. The single-stranded “chair type” 15 mer sequence is d(GGTTGGTGTGGTTGG). The “edge type” 17 mer DNA, d(GGTUTGGTGTGGUTTGG), was also examined [2]. The 15 mer fully forms the quadruplex structure at this KCl concentration [2, 23, 24].

13 nm gold nanoparticles were synthesized by citrate reduction of HAuCl_4 _(tetrachloroauric acid) [32]. Both sodium citrate and tetrachloroauric acid were purchased from Sigma-Aldrich and used without further purification.

### 2.2. Sample Preparation

Samples containing the double-stranded “basket type” 12 mer d(G_4_T_4_G_4_), triplet repeat d(GGC)_3_, 15 mer with K^+^, and 15 mer without K^+^ were prepared using an approach modified from the one described in the literature [11]. Huang and coworkers used a [DNA] : [Au NP] concentration ratio of 8 : 1 and a DNA concentration of 70 nM in their sample [11]. In this work we decreased the amount of nanoparticles relative to DNA ([DNA]/[Au  NP] = 440) to demonstrate that quadruplex detection is possible with lower nanoparticle concentrations and also started with a lower concentration of DNA (2.1 nM) to determine detection limits.

Each sample contains DNA, HEPES buffer (K^+^ added as needed), and deionized water, totaling a volume of 2.7 mL. For all experiments, 300 *μ*L of nanoparticles were added to bring the total sample volume to 3.0 mL. For solutions without DNA, the DNA was replaced with an equivalent volume of buffer.

In the first series of experiments, nanoparticles were added to the preannealed samples immediately prior to measurement, and samples were mixed thoroughly. PRLS intensities were measured. The DNA samples containing the nanoparticles were then annealed by heating to 80°C followed by slow cooling to room temperature, known as a “slower cool” procedure which results in quadruplex formation in the 12 mer, 15 mer, and triplet repeat DNA [24]. Annealing allows the DNA to explore all possible conformations before finding the most energetically favorable conformation. Prior to annealing, these sequences exist as mixtures of different forms of DNA and therefore are not in 100% quadruplex form. However, the 17 mer forms a mixture of sequences when prepared this way [24].

In a second series of experiments, DNA samples were annealed prior to the addition of nanoparticles. This was done to determine whether or not the prolonged presence of nanoparticles or the annealing in presence of nanoparticles had any effect on the PRLS intensity.

### 2.3. Plasmon Resonance Light Scattering

Plasmon resonance light scattering (PRLS) experiments were carried out by simultaneously scanning the excitation and emission monochromators of a Perkin-Elmer LS50B Luminescence Spectrometer. Gold nanoparticles demonstrated a PRLS peak at 540 nm, and changes to the PRLS intensity at this wavelength were measured upon addition of DNA. Once the peak was determined using a free nanoparticle solution, the PRLS intensity was measured for each sample using the “Read” function of the spectrometer software, where a reading was taken after five seconds. Slit widths were 5.0 nm.

Gold nanoparticles synthesized by citrate reduction repel one another due to the negative citrate ions surrounding each individual nanoparticle. The addition of cations results in the disruption of the “citrate shield” and subsequent aggregation of nanoparticles, which in turn leads to an increase in PRLS intensity. In this work, the presence of cations (Na^+^ and K^+^) was critical for quadruplex formation. Therefore, a separate control experiment was carried out to examine the effect of HEPES buffer with Na^+^ and K^+^ on PRLS intensity. At the concentrations of cations used in this work, there was no PRLS enhancement. Therefore, any enhancement that was observed was due to the presence of DNA.

In order to determine error bars for PRLS measurements, three aqueous gold nanoparticle solutions (no HEPES buffer or DNA) were prepared at the concentration used for the DNA experiments. The PRLS intensity of each solution was measured six times at 540 nm, and the average intensities and standard deviations were calculated. The percent error was determined to be 4%. All observed changes to PRLS were outside this margin of error; therefore the PRLS intensity of each solution was measured once. Also, there was very little variation in the intensity reading. For example, the average PRLS intensity (based on five readings, one reading per second) for a nanoparticle solution with no DNA (slit widths = 2.5 nm) was 52.98 ± 0.83. Therefore, the 4% error was the result of human error in sample preparation rather than instrument error.

### 2.4. DNA Melting Analysis

DNA melting experiments were carried out using a Cary 4000 UV-Vis Spectrophotometer by monitoring the absorbance at 295 nm from 25°C to 95°C [33]. The temperature was increased at a rate of 5°C per minute. DNA concentrations were 1.7 *μ*M in order to obtain optimal absorbance at 295 nm. The DNA to nanoparticle concentration ratio was close to 2000 to avoid detector saturation due to high nanoparticle concentration, even at 295 nm. Duplex behavior of the 12 mer and triplet repeat sequence was monitored at 260 nm. Change in nanoparticle absorbance as a function of temperature was negligible at these wavelengths.

## 3. Results and Discussion

**Figure 1:**
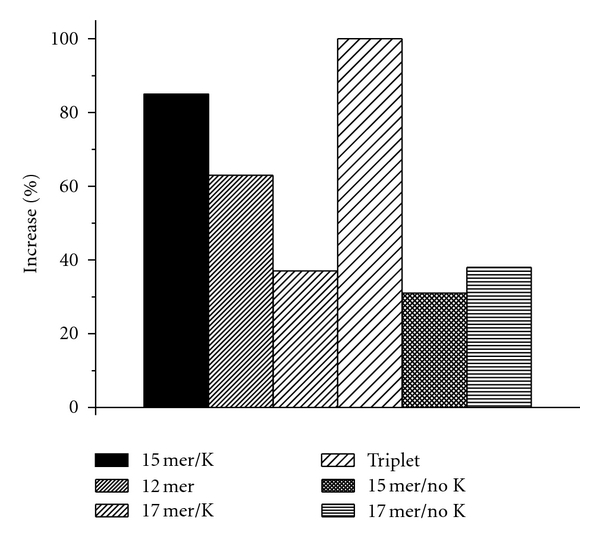
Percentage increase (±4%) in PRLS intensity of gold nanoparticles at 540 nm upon addition of 2.1 nM DNA. Samples were annealed in presence of nanoparticles.

The addition of DNA to the gold nanoparticles resulted in enhancement of the gold nanoparticle PRLS signal only when quadruplex DNA was present (Figure 1). No enhancement was observed when nanoparticles were added to duplex calf thymus DNA. In the quadruplex form, the negatively charged phosphate backbone is exposed to the surrounding liquid, thus inhibiting the interaction of DNA bases with gold nanoparticles and allowing nanoparticle aggregation to take place [11]. Quadruplex sequences form after the DNA has been annealed, and, in certain cases, potassium ions are present.

Figure 1 shows the increase in nanoparticle PRLS intensity at 540 nm when 2.1 nM of each DNA was added and the samples were annealed. Prior to annealing both the triplet repeat DNA and the 15 mer DNA (with K^+^) shown resulted in modest PRLS enhancement, indicating the presence of some quadruplex structure. There was no enhancement observed with the double-stranded 12 mer and the 15 mer without K^+^. This result is consistent with previous findings that potassium is necessary for quadruplex formation [23, 24].

The PRLS intensity increased significantly in presence of the 15 mer (with K^+^), triplet, and 12 mer when the samples were annealed. Annealing is known to facilitate the formation of quadruplex DNA.  Table 1 shows the PRLS enhancement before and after annealing. As two different sample preparation approaches were used, the PRLS enhancement when DNA was annealed in the presence of nanoparticles (shown in Figure 1) and when nanoparticles were added to annealed DNA (not shown) was compared and found to be the same.

Figure 1 also includes results from the 17 mer “edge type” quadruplex sequence. The 17 mer forms a mixture of structures when annealed the same way as the other sequences. PRLS enhancement of 37% was observed in the presence and absence of potassium, which indicates that the different forms present may include structures that are resulting in aggregation of gold nanoparticles, but not to the extent observed with the pure quadruplex sequences (12 mer and 15 mer). 

The key observations from the light scattering experiments are summarized as follows. The single-stranded 15 mer (with K^+^) and triplet repeat DNA enhanced PRLS signal prior to annealing, indicating the presence of some amount of quadruplex sequences. Upon annealing, both the 15 mer (with K^+^) and the triplet repeat DNA resulted in a PRLS enhancement of 85% and 100%, respectively. The results clearly show that the 15 mer requires potassium ions to fully form quadruplex DNA as there was no PRLS enhancement when 15 mer (no K^+^) was added to the gold nanoparticles. However, annealing facilitated some quadruplex formation even in the absence of potassium. Annealing also had a dramatic effect on the double-stranded 12 mer which appeared to form no quadruplex prior to annealing.

In the case of the triplet repeat DNA, the PRLS enhancement before annealing was 18%. Unlike the 15 mer, the triplet repeat DNA has been shown to form a “crossover” type DNA structure in solution [34]. Studies have shown that the d(GGC)_*n*_ sequence, where *n* = 3, 4 or 5, enhances the fluorescence of the duplex DNA intercalator ethidium, indicating that these sequences adopt partial duplex forms [2]. The triplet repeat DNA is also known to form tetrahelical intermolecular structures [35]. Quadruplex DNA causes little-to-modest enhancement of the fluorescence of ethidium. The fact that triplet repeat DNA can also form quadruplex structures at the same time has been shown by its ability to enhance the fluorescence of porphyrins. Another point that must be made is that triplet repeat DNA sequences form stable quadruplex structures at 40 mM potassium concentration [1], which is eightfold higher than the potassium concentration used in the work. Therefore the results obtained under our conditions, which were optimized for the 15 mer DNA, indicate that the triplet repeat DNA, d(GGC)_3_, is forming a mixture of duplex and quadruplex structures and the formation of the latter is leading to the enhanced PRLS intensity prior to annealing.

Duplex DNA, like calf thymus DNA, forms Watson-Crick base pairs which are more stable as shown by the high melting temperature of calf thymus DNA (87°C) but did not enhance the PRLS intensity of gold nanoparticles, even after annealing. The concentration of DNA was increased to 1000 ppb and even then no PRLS enhancement was observed. Therefore, it can be concluded that gold nanoparticles are a good probe for detecting some forms of quadruplex DNA, both partly formed (preannealed with K^+^) and full formed (postannealed) and in mixtures (triplet repeat, 17 mer).

The results presented in Table 1 also show that two different procedures used for sample preparation yielded similar results. The question whether gold nanoparticles are facilitating the formation or stabilization of quadruplex DNA was raised when the enhancement was observed, in the absence of annealing and in the presence of the triplet DNA and 15 mer (with K^+^). On the other hand, the mere presence of K^+^ may be enough to promote quadruplex formation. Since annealed 15 mer (without K^+^) resulted in ~30% enhancement of nanoparticle PRLS; the question that must be asked is, in the absence of potassium, is it the annealing step or the addition of nanoparticles that is facilitating quadruplex formation? This question was easily answered by comparing the PRLS intensity when DNA was annealed in the presence of nanoparticles to the intensity when nanoparticles were added to DNA that had already been annealed (Table 1). When nanoparticles were added to preannealed DNA and the mixture was annealed following PRLS measurement (column 2), the PRLS intensity (column 3) is similar to the intensity measured when gold nanoparticles were added to annealed DNA. This shows that the nanoparticles are unlikely to be facilitating quadruplex formation.

To confirm this, DNA melting experiments were carried out using the DNA samples with and without nanoparticles. Melting transitions involving duplex DNA are typically detected by measuring the change in absorbance at 260 nm, which increases by approximately 25% on denaturation. However, quadruplex DNA shows minimal changes in absorbance at 260 nm. At 295 nm a significantly greater absorbance change is observed, where an increase in absorbance correlates to quadruplex formation, while a decrease in absorbance correlates to DNA melting [33, 36]. The goal of the melting experiments was to determine whether or not the nanoparticles were playing a role in promoting quadruplex formation.

The melting temperature of the 15 mer (35–37°C) did not change even in the absence of K^+^ or annealing. Addition of gold nanoparticles to 15 mer lowered the melting temperature slightly (33°C) even when the samples were annealed but no further quadruplex formation was evident at higher temperatures, indicating that gold nanoparticles may not be promoting further quadruplex formation even in the absence of potassium ions. However, the DNA/nanoparticle concentration ratio was altered for the melting experiments in order to optimize absorbance signal for the DNA and minimize background absorbance by the nanoparticles. Therefore the results of the PRLS experiments cannot be correlated with the melting studies, and this will be the focus of future work. The melting experiments were therefore used to confirm quadruplex or duplex behavior of the various DNA sequences.

To confirm the duplex behavior of the double-stranded 12 mer and the triplet repeat DNA, melting experiments were carried out at 260 nm. The triplet repeat DNA showed significant duplex behavior, with a melting temperature of 80°C. The melting temperature for the 12 mer was 74°C. This shows that the 12 mer and triplet both exhibit duplex behavior, in addition to quadruplex behavior.

### 3.1. Effect of High Temperatures and Aggregation on Nanoparticle Properties

**Figure 2:**
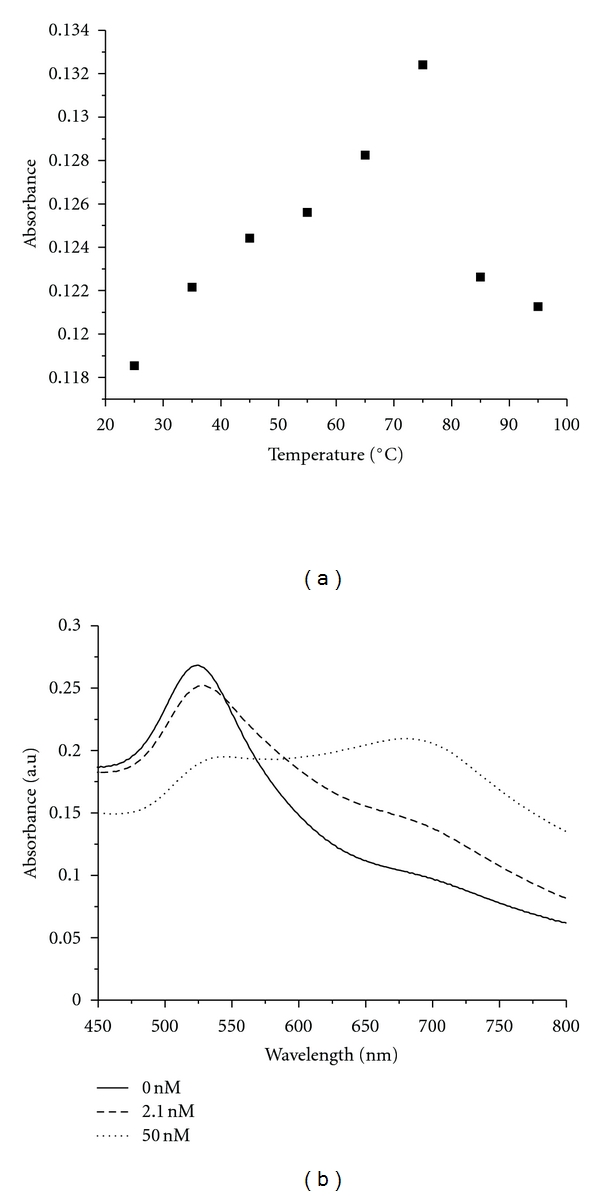
(a) Relationship between gold nanoparticle absorption intensity at 740 nm and temperature. An increase in intensity is observed from 25°C to 75°C, followed by a decrease; (b) change in absorption spectrum of gold nanoparticles as a function of 17 mer DNA concentration.

The change in nanoparticle absorbance at 295 nm as a function of temperature was small but for the PRLS experiments there was a concern that annealing the DNA in presence of nanoparticles would affect the nanoparticles. It has been previously reported that the hydrodynamic diameter of gold colloids increases linearly with temperature until approximately 330 K, where a maximum is obtained [37]. This physical change in the gold nanoparticles is demonstrated by the change in the absorbance at 740 nm, which is higher than the wavelength used for PRLS measurements. The increasing absorbance peak at 740 nm also indicates that larger aggregates (~5/3 larger in diameter than aggregates at 590 nm) were forming at higher temperatures. A sharp decrease in absorbance occurred between 75°C and 85°C, indicating a substantial change in the physical properties of the nanoparticles (Figure 2(a)). While the experiments reported in the literature were carried out with aqueous, salt-free solutions, some of the experiments presented here were carried out in buffered solutions in order to mimic the environment necessary for DNA stability. This difference in ionic strength most likely led to the significantly higher temperature at which the maximum hydrodynamic diameter was observed, and this temperature was slightly lower than the temperature to which the samples were heated for annealing (80°C). Therefore, if future experiments do show that nanoparticles are stabilizing quadruplex sequence or promoting quadruplex formation, the increased aggregation and the presence of larger-sized aggregates may be the likely causes for the increased stabilization of the DNA at higher temperatures in the presence of gold nanoparticles.

Another key observation that was made with regards to nanoparticle properties is the change in color of the solution as the DNA concentration was varied. When 2.1 nM DNA was added, there was an increase in absorption intensity at longer wavelength, indicating that nanoparticle aggregation was taking place (Figure 2(b)). At higher DNA concentration, there was a visible color change in the solution, and the absorbance at 680–700 nm increased significantly.

### 3.2. Concentration Limits

**Figure 3:**
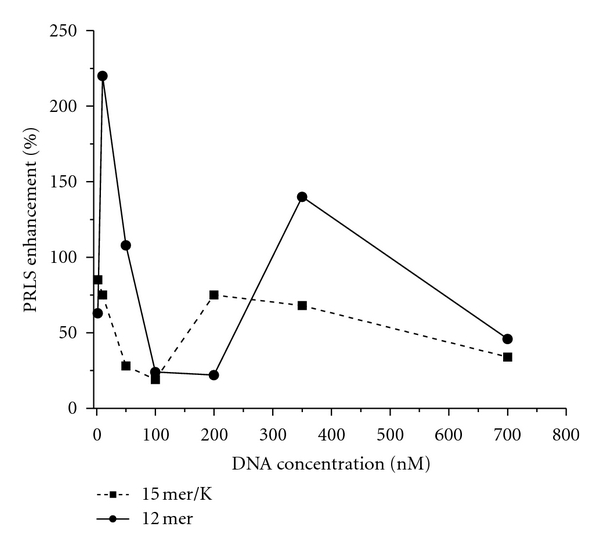
PRLS intensity of gold nanoparticles at 540 nm, measured when different concentrations of 15 mer DNA (with K^+^) and 12 mer were added.

Given that our overall goal is to develop techniques to detect small (nM) amounts of quadruplex DNA using biocompatible probes, PRLS intensities were measured over a wide concentration range (2.1 nM to 700 nM) (Figure 3).

A detection limit is evident. There was a nearly twofold increase in PRLS intensity when the DNA concentration was as low as 2.1 nM (corresponding to 8 ppb 12 mer or 10 ppb 15 mer). The intensity peaked at 10 nM (38 ppb 12 mer, 47 ppb 15 mer) was followed by a sharp decrease and a second increase. A similar trend was observed for the triplet repeat DNA and the 17 mer, as well as the 15 mer and 17 mer without potassium. In our previously published work with lanthanide chelates, we reported that there was also a detection limit (between 20 and 400 ppb for the 15 mer and between 400 and 800 ppb for the 12 mer) [7].

The trend merits further investigation and will be the focus of future work. An equilibrium argument can be explored, as equilibrium exists between the reactants (DNA and free nanoparticles) and the main product (aggregated nanoparticles):

(1) Quadruplex  DNA+free  NPs⇔aggregated  NPs.

However, once equilibrium is reached, the complex concentration should remain constant. Therefore, the decrease in PRLS intensity in the 50–100 nM DNA concentration range might be a result of destabilization of the complex, and the subsequent increase is due to a rearrangement or restructuring that is occurring as more DNA molecules enter the reaction sphere. It must be noted that the PRLS intensity fully recovers for the single-stranded 15 mer quadruplex DNA but not the double-stranded 12 mer DNA, showing the ability of the nanoparticles to distinguish between two different types of quadruplex DNA.

In conclusion, the 8–10 ppb lower limit is lower than the 20 ppb detection limit obtained using terbium chelates [7]. These results indicate that biocompatible probes like nanoparticles can be successful in detecting low concentrations of DNA. The sample preparation is simpler than previously published work, and the samples do not require any special handling. Two different PRLS protocols have been used, and the timing of the nanoparticle addition did not result in a difference in PRLS intensity with annealed DNA. Heating the nanoparticles during the annealing process did not affect the PRLS intensities or enhancement.

## Figures and Tables

**Table 1:** Percent change (±4%) in gold nanoparticle PRLS signal, in presence of preannealed and annealed DNA, and when samples were annealed in the presence of nanoparticles. DNA concentration is 2.1 nM in each case.

DNA	Preannealed	Annealed in presence of AuNPs	Annealed DNA added to AuNPs
15 mer, no K^+^	0	31	29
15 mer, K^+^	26	85	87
Triplet, (GGC)_3_	18	100	95
12 mer	0	63	61

## References

[1] Hardin CC, Watson T, Corregan M, Bailey C (1992). Cation-dependent transition between the quadruplex and Watson-Crick hairpin forms of d(CGCG3GCG). *Biochemistry*.

[2] Arthanari H, Basu S, Kawano TL, Bolton PH (1998). Fluorescent dyes specific for quadruplex DNA. *Nucleic Acids Research*.

[3] Giraldo R, Suzuki M, Chapman L, Rhodes D (1994). Promotion of parallel DNA quadruplexes by a yeast telomere binding protein: a circular dichroism study. *Proceedings of the National Academy of Sciences of the United States of America*.

[4] Drewe WC, Nanjunda R, Gunaratnam M (2008). Rational design of substituted diarylureas: a scaffold for binding to G-quadruplex motifs. *Journal of Medicinal Chemistry*.

[5] Nagatoishi S, Tanaka Y, Tsumoto K (2007). Circular dichroism spectra demonstrate formation of the thrombin-binding DNA aptamer G-quadruplex under stabilizing-cation-deficient conditions. *Biochemical and Biophysical Research Communications*.

[6] Pagba CV, Lane SM, Wachsmann-Hogiu S (2010). Raman and surface-enhanced Raman spectroscopic studies of the 15-mer DNA thrombin-binding aptamer. *Journal of Raman Spectroscopy*.

[7] Worlinsky JL, Basu S (2009). Detection of quadruplex DNA by luminescence enhancement of lanthanide ions and energy transfer from lanthanide chelates. *Journal of Physical Chemistry B*.

[8] Salata O (2004). Applications of nanoparticles in biology and medicine. *Journal of Nanobiotechnology*.

[9] Sperling RA, Casals E, Comenge J, Bastus NG, Puntes VF (2009). Inorganic engineered nanoparticles and their impact on the immune response. *Current Drug Metabolism*.

[10] Sperling RA, Rivera Gil P, Zhang F, Zanella M, Parak WJ (2008). Biological applications of gold nanoparticles. *Chemical Society Reviews*.

[11] Huang CZ, Liao QG, Gan LH, Guo FL, Li YF (2007). Telomere DNA conformation change induced aggregation of gold nanoparticles as detected by plasmon resonance light scattering technique. *Analytica Chimica Acta*.

[12] Aslan K, Lakowicz JR, Geddes CD (2004). Nanogold-plasmon-resonance-based glucose sensing. *Analytical Biochemistry*.

[13] Aslan K, Lakowicz JR, Geddes CD (2005). Nanogold plasmon resonance-based glucose sensing. 2. Wavelength-ratiometric resonance light scattering. *Analytical Chemistry*.

[14] Han H, Hurley LH (2000). G-quadruplex DNA: a potential target for anti-cancer drug design. *Trends in Pharmacological Sciences*.

[15] Burge S, Parkinson GN, Hazel P, Todd AK, Neidle S (2006). Quadruplex DNA: sequence, topology and structure. *Nucleic Acids Research*.

[16] Williamson JR (1994). G-quartet structures in telomeric DNA. *Annual Review of Biophysics and Biomolecular Structure*.

[17] Kim NW, Piatyszek MA, Prowse KR (1994). Specific association of human telomerase activity with immortal cells and cancer. *Science*.

[18] Cosconati S, Marinelli L, Trotta R (2010). Structural and conformational requisites in DNA quadruplex groove binding: another piece to the puzzle. *Journal of the American Chemical Society*.

[19] Hagihara M, Yamauchi L, Seo A, Yoneda K, Senda M, Nakatani K (2010). Antisense-induced guanine quadruplexes inhibit reverse transcription by HIV-1 reverse transcriptase. *Journal of the American Chemical Society*.

[20] Hud NV, Smith FW, Anet FA, Feigon J (1996). The selectivity for K^+^ versus Na^+^ in DNA quadruplexes is dominated by relative free energies of hydration: a thermodynamic analysis by 1 h NMR. *Biochemistry*.

[21] Luu KN, Phan AT, Kuryavyi V, Lacroix L, Patel DJ (2006). Structure of the human telomere in K^+^ solution: an intramolecular (3 + 1) G-quadruplex scaffold. *Journal of the American Chemical Society*.

[22] Williamson JR, Raghuraman MK, Cech TR (1989). Monovalent cation-induced structure of telomeric DNA: the G-quartet model. *Cell*.

[23] Marathias VM, Bolton PH (2000). Structures of the potassium-saturated, 2 : 1, and intermediate, 1 : 1, forms of a quadruplex DNA. *Nucleic Acids Research*.

[24] Marathias VM, Bolton PH (1999). Determinants of DNA quadruplex structural type: sequence and potassium binding. *Biochemistry*.

[25] Wang KY, Krawczyk SH, Bischofberger N, Swaminathan S, Bolton PH (1993). The tertiary structure of a DNA aptamer which binds to and inhibits thrombin determines activity. *Biochemistry*.

[26] Galezowska E, Gluszynska A, Juskowiak B (2007). Luminescence study of G-quadruplex formation in the presence of Tb^3+^ ion. *Journal of Inorganic Biochemistry*.

[27] Feigon J, Dieckmann T, Smith FW (1996). Aptamer structures from A to Zeta. *Chemical and Biology*.

[28] Wang KY, McCurdy S, Shea RG, Swaminathan S, Bolton PH (1993). A DNA aptamer which binds to and inhibits thrombin exhibits a new structural motif for DNA. *Biochemistry*.

[29] Conroy RS, Koretsky AP, Moreland J (2010). Lambda exonuclease digestion of CGG trinucleotide repeats. *European Biophysics Journal*.

[30] Wells RD (1996). Molecular basis of genetic instability of triplet repeats. *Journal of Biological Chemistry*.

[31] Monleón D, Esteve V, Celda B (2003). NMR study of hexanucleotide d(CCGCGG)_2_ containing two triplet repeats of fragile X syndrome. *Biochemical and Biophysical Research Communications*.

[32] Lee PC, Meisel D (1982). Adsorption and surface-enhanced Raman of dyes on silver and gold sols. *The Journal of Physical Chemistry*.

[33] Rachwal PA, Fox KR (2007). Quadruplex melting. *Methods*.

[34] Kettani A, Kumar RA, Patel DJ (1995). Solution structure of a DNA quadruplex containing the fragile X syndrome triplet repeat. *Journal of Molecular Biology*.

[35] Fry M, Loeb LA (1994). The fragile X syndrome D(CGG)_n_ nucleotide repeats form a stable tetrahelical structure. *Proceedings of the National Academy of Sciences of the United States of America*.

[36] Mergny JL, Phan AT, Lacroix L (1998). Following G-quartet formation by UV-spectroscopy. *FEBS Letters*.

[37] Menéndez-Manjón A, Chichkov BN, Barcikowski S (2010). Influence of water temperature on the hydrodynamic diameter of gold nanoparticles from laser ablation. *Journal of Physical Chemistry C*.

